# Students’ perceptions of the educational environment at King Abdulaziz University Faculty of Dentistry (KAUFD): a cross sectional study

**DOI:** 10.1186/s12909-020-02165-7

**Published:** 2020-07-29

**Authors:** Heba J. Sabbagh, Hanin A. Bakhaider, Hesham M. Abokhashabah, Mohammed U. Bader

**Affiliations:** 1 Department of Pediatric Dentistry, King Abdulaziz University, Jeddah, Saudi Arabia; 2 King Abdulaziz University Faculty of Dentistry, Jeddah, Saudi Arabia

**Keywords:** Educational environment, Saudi Arabia, DREEM, Dental students

## Abstract

**Background:**

Prior research studies have found that dental students’ educational environment has an impact on their academic achievement. Therefore, the aim of this cross-sectional study was to assess dental students’ perceptions of the educational environment at King Abdulaziz University Faculty of Dentistry (KAUFD) in Saudi Arabia.

**Methods:**

Second-, third-, and fourth-year dental students at KAUFD, responded to the Dundee Ready Education Environment Measure (DREEM) in October 2017. It consists of five subscales: students’ perceptions of learning, students’ perceptions of teachers, students’ academic self-perceptions, students’ perceptions of the atmosphere, and students’ social self-perceptions. The overall mean value was calculated.

**Results:**

A total of 217 dental students responded to the questionnaire (92 males, 125 females); the response rate was 43.40%. The overall mean DREEM score was 125, which is considered “more positive than negative.” The mean DREEM score was higher for females (128.73) than for males (120.13). Third-year students (137.99) obtained higher mean scores compared to fourth-year (121.42) and fifth-year students (115.94).

**Conclusions:**

Dental students’ perceptions of the educational environment at KAUFD support the findings of national and international studies. Students in the preclinical dental academic year (third year) obtained the highest DREEM score, when compared to those who belonged to the clinical academic years. Therefore, a personal development program and good support systems must be emphasized for clinical-year students.

## Background

Educational environment is a key factor that influences medical students’ education, curriculum satisfaction, learning outcomes, and professional development [1]. Accordingly, improving the educational environment could help establish high-quality university education and promote students’ future success [2, 3]. It is also an effective strategy to measure the efficiency of the university curriculum and student achievement [1]. The Dundee Ready Education Environment Measure (DREEM) was constructed and validated as a universal diagnostic inventory that can assess educational environment. It was originally validated by a Delphi panel that consisted of approximately 100 medical and health educators from several countries [4].

King Abdulaziz University Faculty of Dentistry (KAUFD) in Saudi Arabia has developed strategic goals that help further its mission, namely the achievement of excellence in teaching, learning, and leadership [5]. Their curriculum is described as a traditional curriculum with elements of case-based learning, distributed over a six-year period, with a comprehensive care dental clinic initiated in the sixth year and a 12-month internship. In addition, in the second and third year, preclinical students undergo an orientation week that entails a personal development program (PDP) that is structured to include workshops and lectures on personal growth, mental health, educational skills, goal setting, and self-improvement (including ethics and professionalism) (see Table 1). The PDP was designed to improve the quality of student learning, outcomes, and educational environment. Therefore, the aim of this descriptive analytical cross-sectional study was to assess dental students’ perceptions of the educational environment at KAUFD using the DREEM.

**Table 1:** Distribution of content strategies across the one-week personal development program and its related DREEM subscale

Session	Strategy	DREEM subscale
Session one	Communication & Interpersonal Skills	• Student’s social self-perceptions
Session two	Learning Skills and introduction to courses	• Students’ perception of learning • Students’ academic self-perception
Session three	Practicing and applying ethics/Professional behavior	• Students’ perception of teachers
Session four	Personality development	• students’ perceptions of atmosphere
Session five	Humanistic culture and educational environment	• students’ perceptions of atmosphere

## Methods

Ethical approval for this study was obtained from the ethics committee at KAUFD (047–03-18). All third-, fourth-, and fifth-year dental students, enrolled at KAUFD, were included in this study. The inclusion criterion was students who were attending classes and lectures taught by the dental faculty at the dental school. Students who were pursuing the basic-sciences year and second year of the dental program were excluded because their lectures pertained primarily to medical and basic sciences taught by professors of the Science and Medical college. The sixth year of the program is the final year during which students are typically in the clinics of the University Dental Hospital and attend a limited number of lectures in classrooms. They are fully occupied with comprehensive dental care clinics, presentations related to their clinical cases, and community activity [6].

Students were approached in October 2017. The study setting was designed to assure comfort and reliability to students. Students were introduced to the study face-to-face in their classrooms at noon, after their morning lectures and during their break time, from 12 pm to 1 pm. They were offered a complete verbal explanation of the research methodology and its privacy. They were informed that the study sought their actual opinion and that “truth” is always constructive. They were then invited to participate and sign a consent form for their agreement. The questionnaire was distributed with codes and included no names. The information was gathered from the students through a self-reported questionnaire to allow their free expression. The questionnaire began with students’ demographic data (gender, age, and level of their dental education). To measure student perceptions of the educational environment, the DREEM was used. It consists of five subscales:

- Students’ perceptions of learning: 12 items, max score 48, and interpreted as follows: 0–12, very poor; 13–24, teaching is viewed negatively; 25–36, a more positive perception; 37–48, teaching highly thought of.
- Students’ perceptions of teachers: 11 items, max score 44, and interpreted as follows: 0–11, abysmal; 12–22, in need of some retraining; 23–33, moving in the right direction; and 34–44, model teachers.
- Students’ academic self-perceptions: 8 items, max score 32, and interpreted as follows: 0–8, feeling of total failure; 9–16, many negative aspects; 17–24, feeling more on the positive side; and 25–32, confident.
- Students’ perception of atmosphere: 12 items, max score 48, and interpreted as follows: 0–12, a terrible environment; 13–24, there are many issues that need to be changed; 25–36, a more positive atmosphere; and 37–48, a good feeling overall.
- Students’ social self-perceptions: 7 items, max score 28, and interpreted as follows: 0–7, miserable; 8–14, not a nice place; 15–21, not very bad: and 22–28, very good socially.

In addition, each subscale includes items that record responses on a 5-point Likert scale ranging from “strongly disagree” (score = 0) to “strongly agree” (score = 4). However, nine of the questions were negative statements and were conversely scored. Means and standard deviations (SD) were calculated for each individual item. Individual item values equal to or greater than 3.5 are indicative of a strong presence of the respective aspect of the educational environment, defined as “especially strong,” whereas values that are equal to or less than 2 indicate that the respective aspect requires special attention, defined as “to need particular attention”; mid-range values indicate that the respective aspect of the educational environment offers scope for improvement, defined as “could be improved.”

Moreover, the overall mean value was calculated. A total DREEM score between 151 and 200 is considered excellent. A score between 101 and 150 is considered more positive than negative. A score below 151 is considered as “plenty of problems” or very poor [7–9].

Statistical analyses were conducted using the Statistical Package for Social Sciences v 16.0 (SPSS Inc., Chicago, IL, USA). T-test and ANOVA were used to calculate statistically significant differences. The Bonferroni correction was used to counteract for multiple comparisons. Significance level was set to 0.05.

## Results

Out of a total of 500 third, fourth, and fifth-year dental students, 217 responded to the questionnaire (92 males, 125 females); thus, the response rate was 43.40% (see Table 2). The Cronbach’s alpha analysis for overall DREEM was 0.93, suggesting relatively high internal consistency.

**Table 2:** Distribution of the sample according to academic year and sex

Level	Male	female	***P*** value
**3rd year**	27 (29.34)	50 (40.0)	0.194
**4th year**	33 (35.87)	33 (26.4)	
**5th year**	32 (34.78)	42 (33.6)	
**Total**	**92 (100)**	**125 (100)**	**217**

The overall mean DREEM score was 125, which is considered “more positive than negative.” The mean DREEM score was higher for females (128.73) than for males (120.13). Third-year students (137.99) obtained higher mean scores compared to fourth-year (121.42) and fifth-year students (115.94).

In addition, regarding the mean total scores that were computed for each subscale, students’ perceptions of learning scored 30.4, interpreted as “a more positive approach”; their perception of teachers scored 23.6, interpreted as “moving in the right direction”; their academic self-perception scored 21.7, interpreted as “feeling more on the positive side”; their perception of atmosphere scored 30.4, interpreted as “a more positive atmosphere”; and their social self-perception scored 19.1, interpreted as “not very bad.” Third-year students obtained the highest mean scores for all DREEM subscale items (32.29 for students’ perceptions of learning, 25.97 for students’ perceptions of teachers, 23.77 for students’ academic self-perceptions, 34.31 for atmosphere perceptions, and 21.65 for students’ social self-perceptions) followed by fourth- and fifth-year students (see Table 3).

**Table 3:** Means for the 50 items of the DREEM as a function of academic year and sex

DREEM Items	Male Mean (SD)	female Mean (SD)	***P*** value	Third year Mean (SD)	Fourth year Mean (SD)	Fifth year Mean (SD)	***P*** value
**I. Student’s perceptions of learning** (Max 48)							
**I am encouraged to participate in class**	2.59 (1.187)	2.58 (.835)	0.983	2.70 (1.113)	2.58 (0.895)	2.47 (0.954)	0.372
**The teaching concerned to develop my confidence**	2.63 (1.116)	2.61 (.932)	0.872	2.88 (0.973)	2.62 (1.004)	2.34 (0.997)	0.004*
**The teaching encourages me to be active learner**	2.60 (1.110)	2.75 (.886)	0.257	2.95 (1.012)	2.70 (0.928)	2.41 (0.950)	0.003*
**The teaching is well focused**	2.62 (1.098)	2.98 (.893)	0.009*	3.14 (0.942)	2.71 (1.034)	2.59 (0.950)	0.002*
**The teaching concerned to develop my competence**	2.58 (1.092)	2.74 (.941)	0.226	2.95 (0.916)	2.59 (1.007)	2.46 (1.049)	0.008*
**I am clear about the course learning objectives**	2.58 (1.122)	2.78 (1.077)	0.186	3.03 (1.051)	2.74 (0.917)	2.30 (1.179)	< 0.001
**The teaching is often stimulating**	2.47 (1.032)	2.62 (.811)	0.236	2.74 (0.979)	2.67 (0.829)	2.26 (0.845)	0.002*
**The teaching time is put to good use**	2.25 (1.210)	2.58 (1.049)	0.031*	2.81 (1.014)	2.36 (1.185)	2.14 (1.102)	0.001*
**The teaching is student-centered**	2.46 (1.042)	2.58 (.854)	0.355	2.86 (0.884)	2.42 (0.993)	2.27 (0.849)	< 0.001*
**Long-term learning is emphasized over the short term**	2.8 (0.92)	2.55 (1.17)	0.51	2.15 (1.18)	3.1 (0.89)	3.1 (1.21)	0.031*
**The teaching is too teacher-centered**^**a**^	1.9 (0.88)	1.55 (1.1)	0.325	1.38 (0.96)	1.4 (1.001)	2.03 (1.18)	0.036*
**The teaching overemphasizes factual learning**^**a**^	2.21 (0.79)	2.68 (0.87)	0.096	2.7 (0.82)	2.55 (0.93)	2.8 (0.87)	0.033*
**Total**	**29.69**	**31.1**		**32.29**	**30.44**	**29.17**	
**II. Student’s perceptions of teachers** (Max 44)**:**							
**The teacher providing** feedback to **student**	2.47 (1.133)	2.62 (.965)	0.299	3.03 (1.000)	2.29 (1.049)	2.30 (0.903)	< 0.001*
**The teachers have good communications skills with patients**	2.5 (0.97)	2.62 (0.85)	0.66	2.5 (0.79)	2.6 (0.84)	2.7 (0.97)	0.884
**The teachers give clear examples**	2.65 (.966)	2.93 (.938)	0.041	3.14 (0.854)	2.73 (0.851)	2.53 (1.050)	< 0.001*
**The teachers prepared for their classes**	2.89 (1.114)	3.09 (1.008)	0.176	3.47 (0.788)	2.91 (1.077)	2.61 (1.108)	< 0.001*
**The teachers provide constructive criticism**	2.41 (.985)	2.74 (.817)	0.011*	2.73 (0.883)	2.67 (0.883)	2.39 (0.919)	0.054*
**The students irritate the teachers**^**a**^	1.73 (1.017)	2.02 (1.085)	0.049	1.88 (1.051)	1.89 (1.191)	1.91 (0.968)	0.992
**The teachers ridicule the students a**	1.8 (1.03)	2.05 (1.09)	0.5	2.23 (1.135)	1.7 (1.06)	2.07 (0.998)	0.12
**The teachers get angry in class a**	1.4 (0.84)	1.78 (0.94)	0.235	1.74 (1.04)	1.6 (0.814)	1.86 (0.915)	0.56
**The teachers are authoritarian a**	2.1 (1.01)	2.08 (0.97)	0.9	2.13 (0.913)	1.9 (1.03)	2.21 (0.99)	0.45
**The professors considered my role-models**	2.53 (1.253)	2.78 (1.267)	0.132	3.12 (1.100)	2.59 (1.228)	2.28 (1.330)	< 0.001*
**Total**	**22.48**	**24.71**		**25.97**	**22.88**	**22.86**	
**III. Student’s academic self-perceptions** (Max 32)							
**Memorize all I need**	2.48 (1.000)	2.32 (1.082)	0.273	2.66 (0.954)	2.15 (1.056)	2.31 (1.084)	0.010*
**Much of what I must learn seems relevant to a career in dentistry**	2.41 (1.081)	2.94 (.910)	< 0.001*	3.04 (0.979)	2.67 (0.950)	2.43 (1.035)	0.001*
**I feel I am being well prepared for my profession**	2.50 (1.153)	2.90 (.962)	0.005*	3.16 (0.947)	2.68 (1.055)	2.34 (1.037)	< 0.001*
**Last year’s work has been good preparation for this year’s work**	2.29 (1.245)	2.67 (1.076)	0.017*	2.88 (1.026)	2.42 (1.266)	2.20 (1.110)	0.001*
**My problem-solving skills are being developed**	2.67 (1.214)	2.95 (.958)	0.061	3.17 (0.938)	2.65 (1.183)	2.65 (1.052)	0.003*
**I am confident about passing this year**	2.95 (1.142)	3.17 (1.098)	0.149	3.47 (0.912)	3.08 (1.057)	2.66 (1.231)	< 0.001*
**I have learned a lot about empathy in my profession**	2.73 (1.234)	3.02 (1.096)	0.055*	2.99 (1.094)	3.06 (1.080)	2.65 (1.276)	0.076*
**Learning strategies which worked for me before continuing to work for me now**	2.7 (0.675)	2.52 (0.97)	0.315	2.4 (1.14)	2.19 (1.3)	2.8 (0.77)	0.46
**Total**	**20.82**	**22.49**		**23.77**	**20.9**	**20.04**	
**IV. students’ perceptions of atmosphere** (Max 48)							
**Atmosphere during lecture**	2.47 (1.074)	2.62 (1.162)	0.338	3.18 (0.899)	2.24 (1.096)	2.18 (1.090)	< 0.001*
**Able to ask what I want**	2.70 (1.146)	2.92 (1.052)	0.136	3.16 (1.089)	2.71 (0.924)	2.58 (1.170)	0.003*
**Socially comfortable in class**	2.83 (1.135)	2.99 (1.028)	0.262	3.29 (0.958)	2.91 (1.003)	2.55 (1.136)	< 0.001*
**A chance to develop skills**	2.73 (1.080)	2.76 (1.095)	0.832	3.08 (1.133)	2.67 (0.950)	2.47 (1.076)	0.002*
**Atmosphere during tutorials**	2.61 (1.079)	2.51 (1.075)	0.514	2.90 (0.995)	2.47 (0.980)	2.27 (1.150)	0.001*
**The enjoyment outweighs the Stress of studying dentistry**	2.27 (1.351)	2.31 (1.174)	0.815	2.81 (1.124)	2.14 (1.239)	1.91 (1.218)	< 0.001*
**Atmosphere motivate me as a learner**	2.33 (1.101)	2.54 (1.154)	0.162	2.99 (1.032)	2.32 (0.947)	2.01 (1.176)	< 0.001*
**I can concentrate well**	2.50 (1.074)	2.62 (.990)	0.412	2.99 (0.866)	2.33 (1.086)	2.34 (0.997)	< 0.001*
**Atmosphere during ward**	2.60 (.984)	2.61 (.975)	0.940	2.88 (0.873)	2.47 (0.996)	2.43 (1.008)	0.007*
**This school is well timetabled**	2.42 (1.277)	2.62 (1.037)	0.205	2.81 (1.064)	2.45 (1.205)	2.34 (1.138)	0.033*
**Experience disappointing**	2.09 (1.315)	2.44 (1.153)	0.037*	2.78 (1.382)	2.08 (1.114)	1.97 (1.006)	< 0.001*
**Cheating is a problem in this school**^**a**^	1.39 (1.490)	1.86 (1.376)	0.017*	1.44 (1.381)	1.89 (1.50)	1.69 (1.433)	0.171*
**Total**	**28.94**	**30.8**		**34.31**	**28.68**	**26.74**	
**V. Student’s social self-perceptions** (Max 28)							
**I have good friends in this school**	3.11 (1.320)	3.30 (1.010)	0.222	3.47 (0.887)	3.41 (1.095)	2.80 (1.324)	< 0.001*
**Good support system who get stressed**	1.98 (1.367)	2.18 (1.240)	0.249	2.64 (1.157)	1.79 (1.283)	1.81 (1.279)	< 0.001*
**Too tired to enjoy the course**^**a**^	1.48 (1.2)	1.63 (1.051)	0.317	1.9 (1.095)	1.29 (1.092)	1.47 (1.088)	0.003*
**Rarely bored on course**	2.04 (1.109)	1.92 (1.090)	0.414	2.09 (1.090)	2.02 (1.074)	1.81 (1.119)	0.273
**My accommodation is pleasant**	2.46 (1.073)	2.58 (.909)	0.377	2.81 (1.052)	2.47 (0.964)	2.28 (0.852)	0.004*
**My social life is good**	2.39 (1.374)	2.75 (1.133)	0.036*	2.81 (1.148)	2.61 (1.402)	2.38 (1.190)	0.111
**I seldom feel lonely**	2.34 (1.122)	2.30 (1.157)	0.794	2.44 (1.106)	2.41 (1.176)	2.09 (1.125)	0.125
**I feel belonging to my school**	2.53 (1.471)	3.10 (1.260)	0.002*	3.49 (0.955)	2.52 (1.481)	2.49 (1.436)	< 0.001*
**Total**	**18.32**	**19.76**		**21.65**	**18.52**	**17.13**	
**Mean total score**	120.93	128.93		137.99	121.42	115.94	

Item record responses on a 5-point Likert scale ranging from “strongly disagree” (score = 0) to “strongly agree” (score = 4)

*P value is significant at 0.05

^a^Questions with negative statement and reversely scored

Moreover, the mean item scores were mostly between 2 and 3.50 across all academic years and genders; these scores were representative of a moderate “could be improved” level. There were statistically significant differences between the three academic years regarding their DREEM item mean scores represented by the ANOVA results (see Table 3) and the Bonferroni correction for multiple comparison (see Supplementary Table 1).

## Discussion

The KAUFD curriculum is oriented toward the mission of achieving excellence in teaching, learning, and leadership [5]. This could not have been established without improving the educational environment of the institute because it is essential for student academic achievement [7]. In this study, the DREEM was used to measure the overall educational environment; we also examined other factors such as academic year and sex that could affect student perception of the educational environment.

In this study, the mean of the composite DREEM scores for the third-, fourth-, and fifth-year dental students was 125. This value is similar to the means obtained in previous national studies conducted in medical and dental schools: Al-Quaseem (M = 102), [10] Al-Madina (M = 106), [11] Riyadh (M = 108.42), [12] and Dammam (M = 112.38) [13]. In addition, comparable studies carried out in other parts of the world have reported similar or greater variability in mean DREEM scores: Iran (99.60), [14] Trinidad and Tobago (109.90), [15] Nigeria (118), Germany (122.95), [16] the United Kingdom (124), [8] and Nepal (130) [17]. A more recent study that assessed the academic environment of a 4 year inquiry-based dental curriculum in the United Kingdom reported a high mean DREEM score of 143.58 [18]. The different lengths of educational years and type of curriculum could have contributed to the different DREEM outcome.

Regarding gender differences, female students obtained a slightly higher mean DREEM score than male students. Females obtained significantly higher mean scores on items that were related to teaching strategies, teachers, and their social lives in school. This finding is consistent with those of past studies [11, 15, 16].

In addition, the mean DREEM score was significantly higher among third-year students, as compared to fourth- and fifth-year students. This finding is consistent with those of previous studies of medical schools in both Spain [19] and Turkey [20]. Specifically, the researchers found that preclinical-year students had more positive perceptions of their educational environment than clinical-year students. This finding is also supported by that of a study of medical students in Imam Abdulrahman Bin Faisal University, Dammam, Saudi Arabia [13], where the highest DREEM score was obtained by third-year students. The researchers speculated that the recency of enrollment might have augmented student motivation as they were still exploring the educational environment. Similarly, with regard to KAUFD, the finding may be attributable to the PDP that students had attended during the first week of their second and third academic years. Table 1 lists the PDP session strategies and their related DREEM subscale.

There is worldwide agreement that PDPs are important because they equip medical students with the skills they need for their academic studies [21]. Moreover, several studies have reported that, in order to be successful in the medical field, students require not only knowledge and clinical skills but also interpersonal and communication skills [22]. In a previous study, a PDP that was similar to the program conducted in the present study (five sessions for 15 h across 5 days) was administered to medical and nursing students in India. The students provided feedback, whereby they evaluated the content and outcomes of the program; the researchers found that the students who had attended the PDP showed improvement in their interpersonal and leadership skills, time management skills, emotional stability, and the ability to cope with stress and have a stable social life [22]. This finding might indicate the need for a PDP as part of students’ curriculum which would be repeated annually. Another recommendation is that teachers should play a more active role in the maintenance of student motivation by implementing ongoing motivational strategies that are structured, organized, and student oriented. Such a contention is supported by findings that teachers’ attitudes and personalities affect student motivation [23]. In addition, Weller (2005) suggested that internal motivation is more important and longer-lasting than external motivation, which requires constant reinforcement [24]. Another important point to consider pertains to the tremendous adverse effect of clinical requirements on the stress levels of dental students who are in the last 2 years of the academic program; this emphasizes the need for a PDP during these years.

Our study explored student perceptions of the educational environment, by assessing the effect of gender and the change from non-clinical to mostly-clinical education years. This finding could be used to assess and contribute to future academic planning worldwide. Accordingly, necessary measures should be taken to improve those aspects of the educational environment that have been found to require particular attention. However, further studies are needed to address the limitations of this study. These include studying factors that could have affected the study outcome, such as student socio-cultural background, attendance, and academic grade. In addition, future case-control studies that assess the PDP’s influence on dental students’ perception of the educational environment compared to students in the clinical years that did not include a PDP is recommended. Moreover, a study that assesses students’ perception of the educational environment in their final year (6th year), which is mainly clinical, is also recommended.

## Conclusion

Dental students’ perceptions of the educational environment at KAUFD support the findings of national and international studies. Students in the preclinical dental academic year (third-year) obtained the highest DREEM score, when compared to those in the clinical academic years. Therefore, a PDP and good support systems must be emphasized for clinical-year students.

## Supplementary information

**Additional file 1 Supplementary Table 1**: Bonferroni correction for statistical differences between the DREEM items scores distributed according to year of graduation.

## Data Availability

Data generated or analyzed during this study are included in this published article.

## Abbreviations

KAUFD: King Abdulaziz university faculty of dentistry
DREEM: Dundee ready education environment measure
PDP: personal development program
