# Supplementary material for: Students’ perceptions of the educational environment at King Abdulaziz University Faculty of Dentistry (KAUFD): a cross sectional study
Source: BMC Med Educ. 2020 Jul 29;20:241. doi: 10.1186/s12909-020-02165-7 (PMC7391502; doi:10.1186/s12909-020-02165-7)
Supplement: Supplementary file 1 — Additional file 1 Supplementary Table 1: Bonferroni correction for statistical differences between the DREEM items scores distributed according to year of graduation. [file 12909_2020_2165_MOESM1_ESM.docx]

Supplementary table 1: Bonferroni correction for statistical differences between the DREEM items scores distributed according to year of graduation

* The mean difference is significant at the 0.05 level.

| **Variable** | **Year level** | | **Mean Difference** | **P value** | **95% Confidence Interval** |
| --- | --- | --- | --- | --- | --- |
| **I. Student’s perceptions of learning** | | | | | |
| **I am encouraged to participate in class** | 3rd | 4th | 0.126 | 1.000 | -0.28 - 0.53 |
|  |  | 5th | 0.228 | 0.483 | -0.16 - 0.62 |
|  | 4th | 3rd | -0.126 | 1.000 | -0.53 - 0.28 |
|  |  | 5th | 0.103 | 1.000 | -0.30 - 0.51 |
|  | 5th | 3rd | -0.228 | 0.483 | -0.62 - 0.16 |
|  |  | 4th | -0.103 | 1.000 | -0.51 - 0.30 |
| **The teaching concerned to develop my confidence** | 3rd | 4th | 0.262 | 0.350 | -0.14 - 0.66 |
|  |  | 5th | 0.545^*^ | 0.003 | 0.16 - 0.93 |
|  | 4th | 3rd | -0.262 | 0.350 | -0.66 - 0.14 |
|  |  | 5th | 0.283 | 0.278 | -0.12 - 0.69 |
|  | 5th | 3rd | -0.545^*^ | 0.003 | -0.93 - -0.16 |
|  |  | 4th | -0.283 | 0.278 | -0.69 - 0.12 |
| **The teaching encourages me to be active learner** | 3rd | 4th | 0.251 | 0.368 | -0.14 - 0.64 |
|  |  | 5th | 0.543^*^ | 0.002 | 0.16 - 0.92 |
|  | 4th | 3rd | -0.251 | 0.368 | -0.64 - 0.14 |
|  |  | 5th | 0.292 | 0.228 | -0.10 - 0.69 |
|  | 5th | 3rd | -0.543^*^ | 0.002 | -0.92 - -0.16 |
|  |  | 4th | -0.292 | 0.228 | -0.69 - 0.10 |
| **The teaching is well focused in school** | 3rd | 4th | 0.431^*^ | 0.027 | 0.04 - 0.82 |
|  |  | 5th | 0.548^*^ | 0.002 | 0.17 - 0.93 |
|  | 4th | 3rd | -0.431^*^ | 0.027 | -0.82 - -0.04 |
|  |  | 5th | 0.118 | 1.000 | -0.28 - 0.52 |
|  | 5th | 3rd | -0.548^*^ | 0.002 | -0.93 - -0.17 |
|  |  | 4th | -0.118 | 1.000 | -0.52- 0.28 |
| **The teaching concerned to develop my competence** | 3rd | 4th | 0.357 | 0.098 | -0.04 - 0.76 |
|  |  | 5th | 0.489^*^ | 0.008 | 0.10 - 0.88 |
|  | 4th | 3rd | -0.357 | 0.098 | -0.76 - 0.04 |
|  |  | 5th | 0.131 | 1.000 | -0.27 - 0.54 |
|  | 5th | 3rd | -0.489^*^ | 0.008 | -0.88 - -0.10 |
|  |  | 4th | -0.131 | 1.000 | -0.54 - 0.27 |
| **I am clear about the course learning objectives** | 3rd | 4th | 0.284 | 0.336 | -0.15 - 0.71 |
|  |  | 5th | 0.729^*^ | <0.001* | 0.31 -1.14 |
|  | 4th | 3rd | -0.284 | 0.336 | -0.71 - 0.15 |
|  |  | 5th | 0.445^*^ | 0.041 | 0.01 - 0.88 |
|  | 5th | 3rd | -0.729^*^ | <0.001* | -1.14 - -0.31 |
|  |  | 4th | -0.445^*^ | 0.041 | -0.88 - -0.01 |
| **The teaching is often stimulating** | 3rd | 4th | 0.074 | 1.000 | -0.29 - 0.43 |
|  |  | 5th | 0.484^*^ | 0.003 | 0.13 - 0.83 |
|  | 4th | 3rd | -0.074 | 1.000 | -0.43 - 0.29 |
|  |  | 5th | 0.410^*^ | 0.021 | 0.05 - 0.77 |
|  | 5th | 3rd | -0.484^*^ | 0.003 | -0.83 - -0.13 |
|  |  | 4th | -0.410^*^ | 0.021 | -0.77 - -0.05 |
| **The teaching time is put to good use** | 3rd | 4th | 0.442 | 0.052 | 0.00 - 0.89 |
|  |  | 5th | 0.670^*^ | 0.001 | 0.24 - 1.10 |
|  | 4th | 3rd | -0.442 | 0.052 | -0.89 - 0.00 |
|  |  | 5th | 0.229 | 0.661 | -0.22 - 0.68 |
|  | 5th | 3rd | -0.670^*^ | 0.001 | -1.10 - -0.24 |
|  |  | 4th | -0.229 | 0.661 | -0.68 - 0.22 |
| **The teaching is student-centered** | 3rd | 4th | 0.433^*^ | 0.015 | 0.07 - 0.80 |
|  |  | 5th | 0.587^*^ | <0.001* | 0.23 - 0.94 |
|  | 4th | 3rd | -0.433^*^ | 0.015 | -0.80 - -0.07 |
|  |  | 5th | 0.154 | 0.952 | -0.22 - 0.52 |
|  | 5th | 3rd | -0.587^*^ | <0.001* | -0.94 - -0.23 |
|  |  | 4th | -0.154 | 0.952 | -0.52 - 0.22 |
| **Ii. Student’s perceptions of teachers** | | | | | |
| **The teacher providing feedback to student** | 3rd | 4th | 0.738^*^ | <0.001* | 0.34 - 1.14 |
|  |  | 5th | 0.729^*^ | <0.001* | 0.34 - 1.11 |
|  | 4th | 3rd | -0.738^*^ | <0.001* | -1.14 - -0.34 |
|  |  | 5th | -0.009 | 1.000 | -0.41 - 0.39 |
|  | 5th | 3rd | -0.729^*^ | <0.001* | -1.11 - -0.34 |
|  |  | 4th | 0.009 | 1.000 | -0.39 - 0.41 |
| **The teachers give clear examples** | 3rd | 4th | 0.416^*^ | 0.024 | 0.04 - 0.79 |
|  |  | 5th | 0.616^*^ | <0.001* | 0.25 - 0.98 |
|  | 4th | 3rd | -0.416^*^ | 0.024 | -0.79 - -0.04 |
|  |  | 5th | 0.200 | 0.607 | -0.18 - 0.58 |
|  | 5th | 3rd | -0.616^*^ | <0.001* | -0.98 - -0.25 |
|  |  | 4th | -0.200 | 0.607 | -0.58 - 0.18 |
| **The teachers prepared for their classes** | 3rd | 4th | 0.558^*^ | 0.003 | 0.16 - 0.96 |
|  |  | 5th | 0.859^*^ | <0.001* | 0.47 -1.25 |
|  | 4th | 3rd | -0.558^*^ | 0.003 | -0.96 - -0.16 |
|  |  | 5th | 0.301 | 0.227 | -0.11 - 0.71 |
|  | 5th | 3rd | -0.859^*^ | <0.001* | -1.25 - -0.47 |
|  |  | 4th | -0.301 | 0.227 | -0.71 -0.11 |
| **The teachers provide constructive criticism** | 3rd | 4th | 0.061 | 1.000 | -0.30 - 0.42 |
|  |  | 5th | 0.335 | 0.067 | -0.02 - 0.69 |
|  | 4th | 3rd | -0.061 | 1.000 | -0.42 - 0.30 |
|  |  | 5th | 0.275 | 0.214 | -0.09 - 0.64 |
|  | 5th | 3rd | -0.335 | 0.067 | -0.69 - 0.02 |
|  |  | 4th | -0.275 | 0.214 | -0.64 - 0.09 |
| **The student irritates the teachers** | 3rd | 4th | 0.011 | 1.000 | -0.42 - 0.44 |
|  |  | 5th | 0.022 | 1.000 | -0.40 - 0.44 |
|  | 4th | 3rd | -0.011 | 1.000 | -0.44 - 0.42 |
|  |  | 5th | 0.011 | 1.000 | -0.43 - 0.45 |
|  | 5th | 3rd | -0.022 | 1.000 | -0.44 - 0.40 |
|  |  | 4th | -0.011 | 1.000 | -0.45 - 0.43 |
| **Iii. Student’s academic self-perceptions** | | | | | |
| **Memorize all I need** | 3rd | 4th | 0.511^*^ | 0.010 | 0.09 - 0.93 |
|  |  | 5th | 0.352 | 0.112 | -0.05 - 0.76 |
|  | 4th | 3rd | -0.511^*^ | 0.010 | -0.93 - -0.09 |
|  |  | 5th | -0.159 | 1.000 | -0.58 - 0.26 |
|  | 5th | 3rd | -0.352 | 0.112 | -0.76 - 0.05 |
|  |  | 4th | 0.159 | 1.000 | -0.26 - 0.58 |
| **Much of what I have to learn seems relevant to a career in medicine** | 3rd | 4th | 0.372 | 0.078 | -0.03 - 0.77 |
|  |  | 5th | 0.607^*^ | 0.001 | 0.22 - 1.00 |
|  | 4th | 3rd | -0.372 | 0.078 | -0.77 - 0.03 |
|  |  | 5th | 0.234 | 0.491 | -0.17 - 0.64 |
|  | 5th | 3rd | -0.607^*^ | 0.001 | -1.00 - -0.22 |
|  |  | 4th | -0.234 | 0.491 | -0.64 - 0.17 |
| **I feel I am being well prepared for my profession** | 3rd | 4th | 0.474^*^ | 0.017 | 0.06 - 0.88 |
|  |  | 5th | 0.818^*^ | <0.001* | 0.42 - 1.22 |
|  | 4th | 3rd | -0.474^*^ | 0.017 | -0.88 - -0.06 |
|  |  | 5th | 0.344 | 0.138 | -0.07 - 0.76 |
|  | 5th | 3rd | -0.818^*^ | <0.001* | -1.22 - -0.42 |
|  |  | 4th | -0.344 | 0.138 | -0.76 - 0.07 |
| **Last year’s work has been good preparation for this year’s work** | 3rd | 4th | 0.459^*^ | 0.049 | 0.00 - 0.92 |
|  |  | 5th | 0.680^*^ | 0.001 | 0.24 - 1.12 |
|  | 4th | 3rd | -0.459^*^ | 0.049 | -0.92 - 0.00 |
|  |  | 5th | 0.222 | 0.746 | -0.24 - 0.68 |
|  | 5th | 3rd | -0.680^*^ | 0.001 | -1.12 - -0.24 |
|  |  | 4th | -0.222 | 0.746 | -0.68 - 0.24 |
| **My problem-solving skills are being developed** | 3rd | 4th | 0.517^*^ | 0.012 | 0.09 - 0.94 |
|  |  | 5th | 0.520^*^ | 0.008 | 0.11 - 0.93 |
|  | 4th | 3rd | -0.517^*^ | 0.012 | -0.94 - -0.09 |
|  |  | 5th | 0.003 | 1.000 | -0.43 - 0.43 |
|  | 5th | 3rd | -0.520^*^ | 0.008 | -0.93 - -0.11 |
|  |  | 4th | -0.003 | 1.000 | -0.43 - 0.43 |
| **I am confident about passing this year** | 3rd | 4th | 0.392 | 0.092 | -0.04 - 0.83 |
|  |  | 5th | 0.805^*^ | <0.001* | 0.38 - 1.23 |
|  | 4th | 3rd | -0.392 | 0.092 | -0.83 - 0.04 |
|  |  | 5th | 0.414 | 0.071 | -0.02 - 0.85 |
|  | 5th | 3rd | -0.805^*^ | <0.001* | -1.23 - -0.38 |
|  |  | 4th | -0.414 | 0.071 | -0.85- 0.02 |
| **I have learned a lot about empathy in my profession** | 3rd | 4th | -0.074 | 1.000 | -0.54 - .39 |
|  |  | 5th | 0.338 | 0.220 | -0.12 - 0.79 |
|  | 4th | 3rd | 0.074 | 1.000 | -0.39 - 0.54 |
|  |  | 5th | 0.412 | 0.109 | -0.06 - 0.88 |
|  | 5th | 3rd | -.0338 | 0.220 | -0.79 - 0.12 |
|  |  | 4th | -0.412 | 0.109 | -0.88 - 0.06 |
| **Iv. Students’ perceptions of atmosphere** | | | | | |
| **Atmosphere during lecture** | 3rd | 4th | 0.939^*^ | <0.001* | 0.52 -1.36 |
|  |  | 5th | 1.006^*^ | <0.001* | 0.60 -1.41 |
|  | 4th | 3rd | -0.939^*^ | <0.001* | -1.36 - -0.52 |
|  |  | 5th | 0.067 | 1.000 | -0.35 - 0.49 |
|  | 5th | 3rd | -1.006^*^ | <0.001* | -1.41 - -0.60 |
|  |  | 4th | -0.067 | 1.000 | -0.49 -0.35 |
| **Able to ask what I want** | 3rd | 4th | 0.444^*^ | 0.043 | 0.01 - 0.88 |
|  |  | 5th | 0.575^*^ | 0.003 | 0.15 -1.00 |
|  | 4th | 3rd | -0.444^*^ | 0.043 | -0.88 - -0.01 |
|  |  | 5th | 0.131 | 1.000 | -0.31 -0.57 |
|  | 5th | 3rd | -0.575^*^ | 0.003 | -1.00 - -0.15 |
|  |  | 4th | -0.131 | 1.000 | -0.57 - 0.31 |
| **Socially comfortable in class** | 3rd | 4th | 0.377 | 0.094 | -0.04 - 0.80 |
|  |  | 5th | 0.732^*^ | <0.001* | 0.32 - 1.14 |
|  | 4th | 3rd | -0.377 | 0.094 | -0.80 - 0.04 |
|  |  | 5th | 0.355 | 0.132 | -0.07 - 0.78 |
|  | 5th | 3rd | -0.732^*^ | <0.001* | -1.14 - -0.32 |
|  |  | 4th | -0.355 | 0.132 | -0.78 - 0.07 |
| **A chance to develop skills** | 3rd | 4th | 0.411 | 0.065 | -0.02 - 0.84 |
|  |  | 5th | 0.605^*^ | 0.002 | 0.19 - 1.02 |
|  | 4th | 3rd | -0.411 | 0.065 | -0.84 - 0.02 |
|  |  | 5th | 0.194 | 0.846 | -0.24 - 0.63 |
|  | 5th | 3rd | -0.605^*^ | 0.002 | -1.02 - -0.19 |
|  |  | 4th | -0.194 | 0.846 | 0-.63 - 0.24 |
| **Atmosphere during tutorials**  **-** | 3rd | 4th | 0.426^*^ | 0.048 | 0.00 - 0.85 |
|  |  | 5th | 0.626^*^ | 0.001 | 0.21 - 1.04 |
|  | 4th | 3rd | -0.426^*^ | 0.048 | -0.85 -0.00 |
|  |  | 5th | 0.199 | 0.784 | -0.23 - 0.63 |
|  | 5th | 3rd | -0.626^*^ | 0.001 | -1.04 - -.21 |
|  |  | 4th | -0.199 | 0.784 | -0.63 - 0.23 |
| **The enjoyment outweighs the Stress of studying dentistry** | 3rd | 4th | 0.669^*^ | 0.003 | 0.19 - 1.15 |
|  |  | 5th | 0.900^*^ | <0.001* | 0.43 - 1.37 |
|  | 4th | 3rd | -0.669^*^ | 0.003 | -1.15 - -0.19 |
|  |  | 5th | 0.231 | 0.761 | -0.26 -0.72 |
|  | 5th | 3rd | -0.900^*^ | <0.001* | -1.37 - -0.43 |
|  |  | 4th | -0.231 | 0.761 | -0.72 - 0.26 |
| **Atmosphere motivate me as a learner** | 3rd | 4th | 0.669^*^ | 0.001 | .24 -1.10 |
|  |  | 5th | 0.973^*^ | <0.001* | 0.56 - 1.39 |
|  | 4th | 3rd | -0.669^*^ | 0.001 | -1.10 - -0.24 |
|  |  | 5th | 0.305 | 0.273 | -0.13 - 0.74 |
|  | 5th | 3rd | -0.973^*^ | <0.001* | -1.39 - -0.56 |
|  |  | 4th | -0.305 | 0.273 | -0.74 - 0.13 |
| **I can concentrate well** | 3rd | 4th | 0.654^*^ | <0.001* | 0.26 - 1.05 |
|  |  | 5th | 0.649^*^ | <0.001* | 0.26 - 1.03 |
|  | 4th | 3rd | -0.654^*^ | <0.001* | -1.05 - -0.26 |
|  |  | 5th | -0.005 | 1.000 | -0.41 - 0.40 |
|  | 5th | 3rd | -0.649^*^ | <0.001* | -1.03 - -0.26 |
|  |  | 4th | 0.005 | 1.000 | -0.40 - 0.41 |
| **Atmosphere during ward teaching** | 3rd | 4th | 0.413^*^ | 0.032 | 0.03 - 0.80 |
|  |  | 5th | 0.451^*^ | 0.013 | 0.07 - 0.83 |
|  | 4th | 3rd | -0.413^*^ | .032 | -0.80 - -0.03 |
|  |  | 5th | 0.037 | 1.000 | -0.35 - 0.43 |
|  | 5th | 3rd | -0.451^*^ | 0.013 | -0.83 - -0.07 |
|  |  | 4th | -0.037 | 1.000 | -0.43 - 0.35 |
| **This school is well timetabled** | 3rd | 4th | 0.351 | 0.200 | -0.11 - .081 |
|  |  | 5th | 0.467^*^ | 0.036 | 0.02 - 0.91 |
|  | 4th | 3rd | -0.351 | 0.200 | -0.81 - 0.11 |
|  |  | 5th | 0.117 | 1.000 | -0.35 - 0.58 |
|  | 5th | 3rd | -0.467^*^ | 0.036 | -0.91 - -0.02 |
|  |  | 4th | -0.117 | 1.000 | -0.58 - 0.35 |
| **Reverse experience disappointing** | 3rd | 4th | 0.703^*^ | 0.001 | 0.22 - 1.18 |
|  |  | 5th | 0.806^*^ | <0.001* | 0.34 - 1.27 |
|  | 4th | 3rd | -0.703^*^ | 0.001 | -1.18 - -0.22 |
|  |  | 5th | 0.103 | 1.000 | -0.38 - 0.59 |
|  | 5th | 3rd | -0.806^*^ | <0.001* | -1.27 - -0.34 |
|  |  | 4th | -0.103 | 1.000 | -0.59 - 0.38 |
| **Experience disappointing** | 3rd | 4th | -0.703^*^ | 0.001 | -1.18 - -0.22 |
|  |  | 5th | -0.806^*^ | <0.001* | -1.27 - -0.34 |
|  | 4th | 3rd | 0.703^*^ | 0.001 | 0.22 - 1.18 |
|  |  | 5th | -0.103 | 1.000 | -0.59 - 0.38 |
|  | 5th | 3rd | 0.806^*^ | <0.001* | 0.34 - 1.27 |
|  |  | 4th | 0.103 | 1.000 | -0.38 - 0.59 |
| **Cheating is a problem in this school** | 3rd | 4th | -0.452 | 0.185 | -1.03 - 0.13 |
|  |  | 5th | -0.248 | 0.872 | -0.81 - 0.32 |
|  | 4th | 3rd | 0.452 | 0.185 | -0.13 - 1.03 |
|  |  | 5th | 0.205 | 1.000 | -0.38 - 0.79 |
|  | 5th | 3rd | 0.248 | 0.872 | -0.32 - 0.81 |
|  |  | 4th | -0.205 | 1.000 | -0.79 - 0.38 |
| **V. Student’s social self-perceptions** | | | | | |
| **I have good friends in this school** | 3rd | 4th | 0.065 | 1.000 | -0.39 - 0.52 |
|  |  | 5th | 0.676^*^ | 0.001 | 0.24 - 1.12 |
|  | 4th | 3rd | -0.065 | 1.000 | -0.52 - 0.39 |
|  |  | 5th | 0.612^*^ | 0.004 | 0.16 - 1.07 |
|  | 5th | 3rd | -0.676^*^ | 0.001 | -1.12 - -0.24 |
|  |  | 4th | -0.612^*^ | 0.004 | -1.07 - -0.16 |
| **Good support system who get stressed** | 3rd | 4th | 0.848^*^ | <0.001* | 0.35 - 1.35 |
|  |  | 5th | 0.826^*^ | <0.001* | 0.34 - 1.31 |
|  | 4th | 3rd | -0.848^*^ | <0.001* | -1.35 - 0.35 |
|  |  | 5th | -0.023 | 1.000 | -0.53 - 0.48 |
|  | 5th | 3rd | -0.826^*^ | <0.001* | -1.31 - -0.34 |
|  |  | 4th | 0.023 | 1.000 | -0.48 - 0.53 |
| **Too tired to enjoy the course** | 3rd | 4th | -0.608^*^ | 0.003 | -1.05 - -0.17 |
|  |  | 5th | -0.423 | 0.055 | -0.85-0.01 |
|  | 4th | 3rd | 0.608^*^ | 0.003 | 0.17 - 1.05 |
|  |  | 5th | 0.185 | 0.954 | -0.26 - 0.63 |
|  | 5th | 3rd | 0.423 | 0.055 | 0.00 - 0.85 |
|  |  | 4th | -0.185 | 0.954 | -0.63 - 0.26 |
| **Rarely bored on course** | 3rd | 4th | 0.076 | 1.000 | -0.37 - 0.52 |
|  |  | 5th | 0.280 | 0.353 | -0.15 - 0.71 |
|  | 4th | 3rd | -0.076 | 1.000 | -0.52 - 0.37 |
|  |  | 5th | 0.204 | 0.815 | -0.24 - 0.65 |
|  | 5th | 3rd | -0.280 | 0.353 | -0.71 - 0.15 |
|  |  | 4th | -0.204 | 0.815 | -0.65 - 0.24 |
| **My accommodation is pleasant** | 3rd | 4th | 0.335 | 0.116 | -0.05 - 0.72 |
|  |  | 5th | 0.521^*^ | 0.003 | 0.14 - 0.90 |
|  | 4th | 3rd | -0.335 | 0.116 | -0.72 - 0.05 |
|  |  | 5th | 0.186 | 0.763 | -0.21 - 0.58 |
|  | 5th | 3rd | -0.521^*^ | 0.003 | -0.90 - -0.14 |
|  |  | 4th | -0.186 | 0.763 | -0.58 - 0.21 |
| **My social life is good** | 3rd | 4th | 0.199 | 1.000 | -0.30 - 0.70 |
|  |  | 5th | 0.427 | 0.109 | -0.06 - 0.92 |
|  | 4th | 3rd | -0.199 | 1.000 | -0.70 - 0.30 |
|  |  | 5th | 0.228 | 0.843 | -0.28 - 0.74 |
|  | 5th | 3rd | -0.427 | 0.109 | -0.92 - 0.06 |
|  |  | 4th | -0.228 | 0.843 | -0.74 - 0.28 |
| **I seldom feel lonely** | 3rd | 4th | 0.032 | 1.000 | -0.43 - 0.49 |
|  |  | 5th | 0.347 | 0.185 | -0.10 - 0.79 |
|  | 4th | 3rd | -0.032 | 1.000 | -0.49 - 0.43 |
|  |  | 5th | 0.314 | 0.309 | -0.15 - 0.78 |
|  | 5th | 3rd | -0.347 | 0.185 | -0.79 - 0.10 |
|  |  | 4th | -0.314 | 0.309 | -0.78 - 0.15 |
